# Nerve trunk healing and neuroma formation after nerve transection injury

**DOI:** 10.3389/fneur.2023.1184246

**Published:** 2023-06-12

**Authors:** Dong-Xu Huang, Ming-Xi Yang, Zhen-Min Jiang, Miao Chen, Kun Chang, Yong-Xin Zhan, Xu Gong

**Affiliations:** Department of Hand and Foot Surgery, Orthopaedics Center, The First Hospital of Jilin University, Changchun, Jilin, China

**Keywords:** peripheral nerve injury, angiogenesis, nerve fiber regeneration, scarring, traumatic neuroma, protein kinase inhibitor

## Abstract

The nerve trunk healing process of a transected peripheral nerve trunk is composed of angiogenesis, nerve fiber regeneration, and scarring. Nerve trunk healing and neuroma formation probably share identical molecular mediators and similar regulations. At the nerve transection site, angiogenesis is sufficient and necessary for nerve fiber regeneration. Angiogenesis and nerve fiber regeneration reveal a positive correlation in the early time. Scarring and nerve fiber regeneration show a negative correlation in the late phase. We hypothesize that anti-angiogenesis suppresses neuromas. Subsequently, we provide potential protocols to test our hypothesis. Finally, we recommend employing anti-angiogenic small-molecule protein kinase inhibitors to investigate nerve transection injuries.

## 1. Introduction

Nowadays, advances in experimental investigations have not significantly influenced their clinical applications in the research field of peripheral nerve injury (1–4). The nerve fiber regeneration does not always parallel the neural function recovery when the nerve trunk is completely transected. Each transected peripheral axon in laboratory animals regenerates robustly, but none of the severed human nerves restore their functions completely. Nerve transection injuries represent the most challenging and common situation (5). Nerve crush injuries occupy only a tiny proportion (6). Therefore, basic or applied investigations using nerve transection models may be more meaningful than nerve crush models.

In this review, we will propose the concept of nerve trunk healing, a neurobiological process following nerve transection injury. Subsequently, we will raise several questions and try to answer them. What is the relationship between nerve trunk healing and neuroma formation? What are the relationships among angiogenesis, nerve fiber regeneration, and scarring at the nerve transection sites? Inspired by the anti-angiogenic strategy in tumor therapy, we will hypothesize that anti-angiogenesis can suppress neuromas. Subsequently, we will provide protocols to verify our hypothesis. We will end this review by highlighting the potential repurposing of the commercially-approved anti-angiogenic small-molecule protein kinase inhibitors in the field of peripheral nerve injury.

## 2. The nerve trunk and the surrounding tissue bed

**Figure 1:**
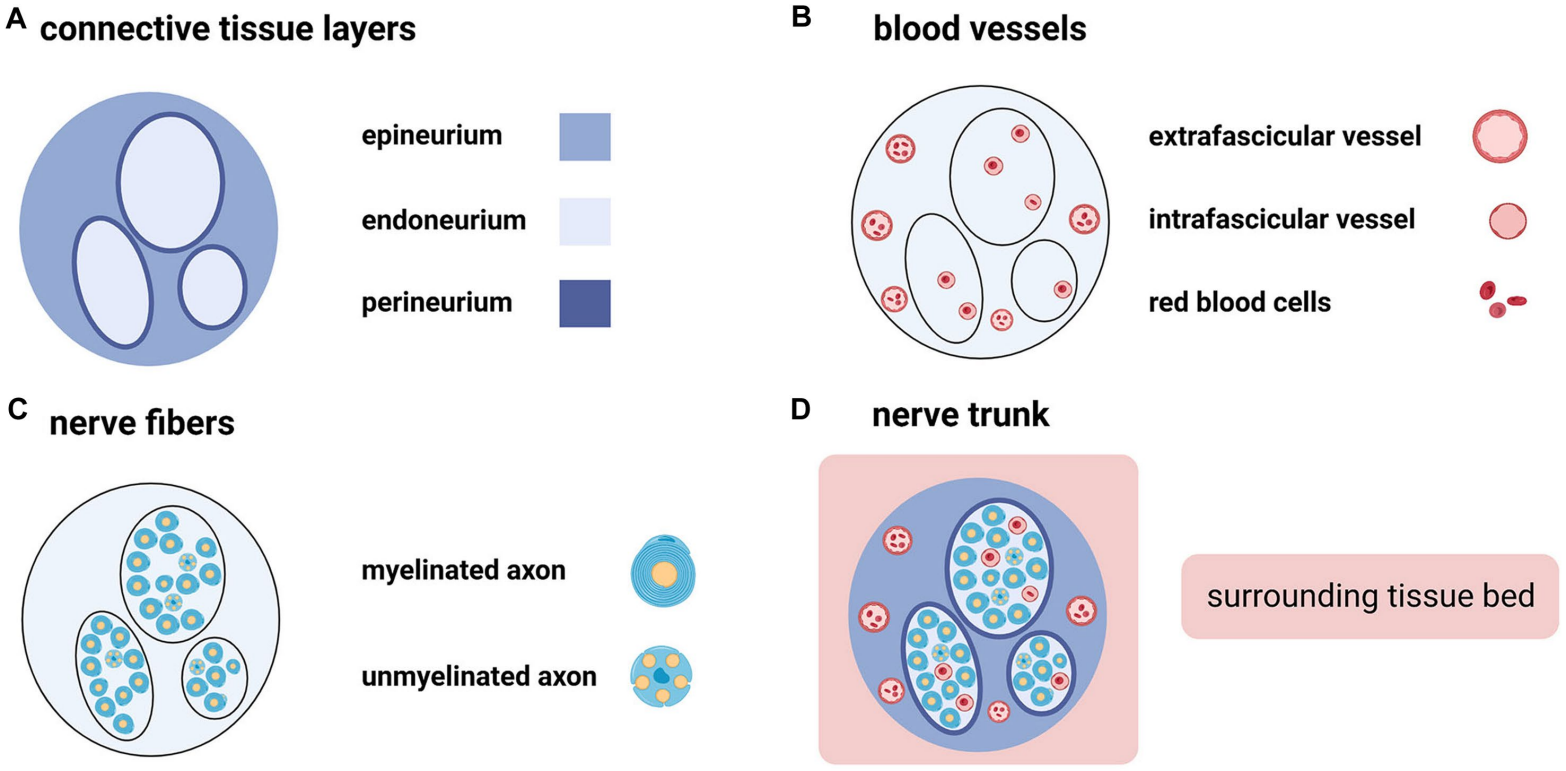
The peripheral nerve trunk consists of connective tissue layers, blood vessels, and nerve fibers (Created with BioRender.com). **(A)** Connective tissue components of the peripheral nerve trunk are arranged into three intimately connected layers from the outside to the inside: epineurium, perineurium, and endoneurium. **(B)** Extrafascicular and intrafascicular vessels spread throughout the epineurium and endoneurium, respectively. **(C)** Nerve fibers include myelinated axons and unmyelinated axons. **(D)** The peripheral nerve trunk is embedded in the surrounding tissue bed.

As a finely-organized three-dimensional structure, the peripheral nerve trunk consists of not only nerve fibers but also connective tissues and blood vessels (Figure 1) (7). An intact nerve trunk lies on the “surrounding tissue bed” (8, 9). It may comprise muscles, tendons, fasciae, blood vessels, or fats. Nerve trunks are not fixed but shift in the surrounding tissue bed (10). Connective tissue components of the peripheral nerve trunk are arranged into the endoneurium, perineurium, and epineurium (Figure 1) (11, 12). Like skin to the body, the epineurium is the edge of a nerve trunk (11). Outside the epineurium is the paraneurium (or mesoneurium), where the nerve trunk glide (8, 11, 13). A peripheral nerve trunk owns two microvascular systems: the extraneural “extrinsic system” and the intraneural “intrinsic system” (Figure 1) (14). The former spreads in the paraneurium (14); the latter spreads throughout the three connective tissue layers (15).

Taken together, a nerve trunk seems like an organ, rather than a single tissue, which slides in the surrounding tissue bed and transmits electrical impulses: (i) its parenchyma comprises both myelinated and unmyelinated nerve fibers (peripheral axons and Schwann cell sheath); (ii) its stroma consists of epineurium, perineurium, and endoneurium as well as blood vessels; and (iii) Schwann cell basal laminae separate the parenchyma from the stroma.

## 3. Changes of nerve trunks after transection injury

### 3.1. Interstump gaps in which “new structures” form

**Figure 2:**
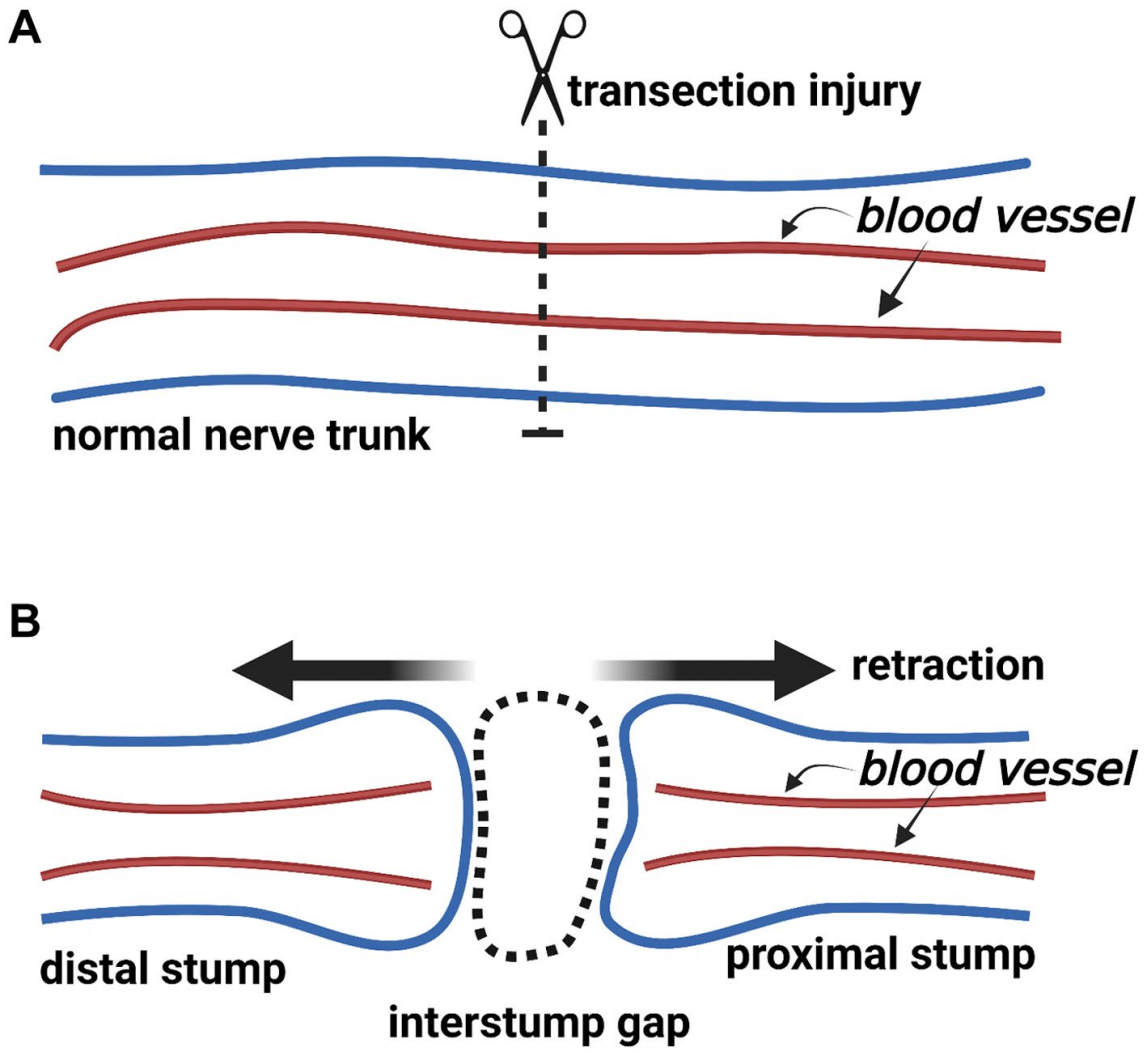
The change of a nerve trunk after transection injury (Created with BioRender.com). **(A)** The normal nerve trunk and extrafascicular blood vessels. **(B)** After nerve transection injury, the nerve trunk divides into the proximal and distal stumps. Two stumps retract because of the elasticity within the nerve trunk. An interstump gap inevitably forms.

**Figure 3:**
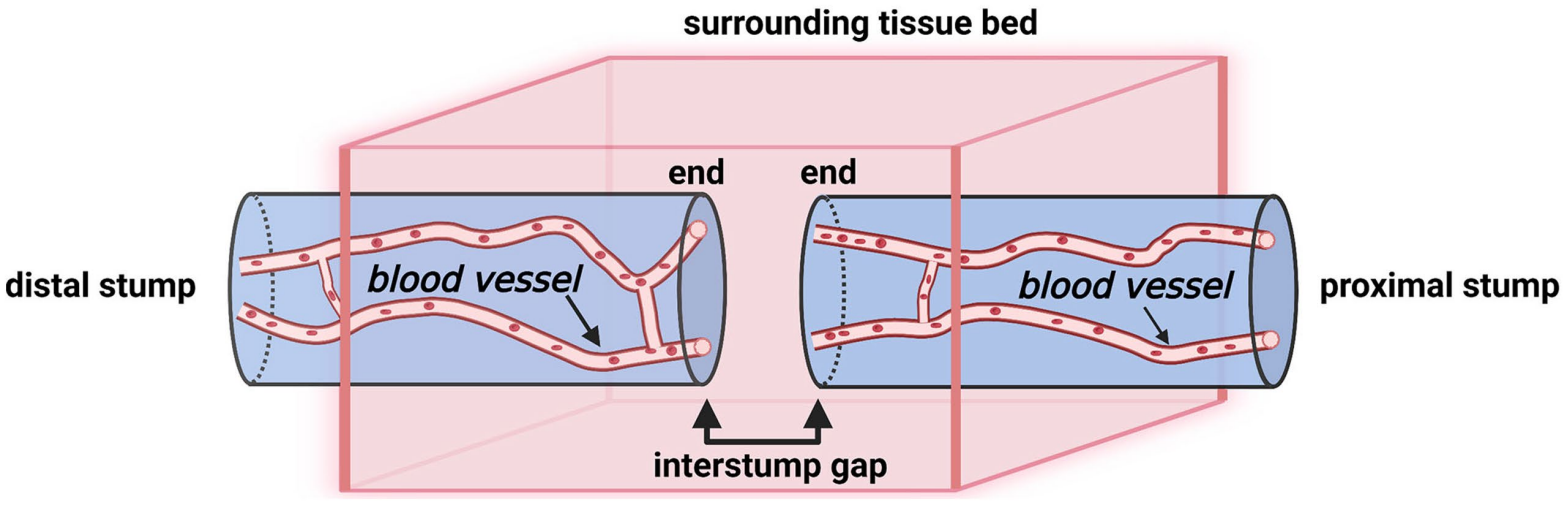
A schematic illustration of a transected nerve trunk and its surrounding tissue bed (Created with BioRender.com). The nerve transection site has four individual compartments: the end of the proximal stump, the interstump gap, the end of the distal stump, and the surrounding tissue bed.

The nerve trunk divides into the proximal and distal stumps following a complete transection injury. Because of the elasticity within the nerve trunk, both stumps retract (Figure 2) (16). Subsequently, the so-called “interstump gap” named by Göran Lundborg inevitably forms between the ends of two stumps (5). Hence, we consider that there are four compartments at the nerve transection site: (i) the interstump gap, (ii) the end of the proximal stump, (iii) the end of the distal stump, and (iv) the surrounding tissue bed (Figure 3).

These interstump gaps are biological battlefields where axons and multiple cell types interact, such as Schwann cells, macrophages, endothelial cells, and fibroblasts (5). These cells originate mainly from the proximal and distal stumps (17–25). In animal models, under an appropriate length of the interstump gap, these cells can spontaneously span the interstump gap and subsequently form a “new structure.” The “new structure” is named differently in several animal experiments with different study designs, such as “neuromas” (26–29), “suture line neuromas” (30, 31), “neuroma scars” (32), and “bridges” (33, 34) (also see Section 3.4). Despite various nomenclatures for the “new structure,” the common result is the uniting of two nerve stumps.

### 3.2. The nerve trunk healing process at nerve transection sites

**Figure 4:**
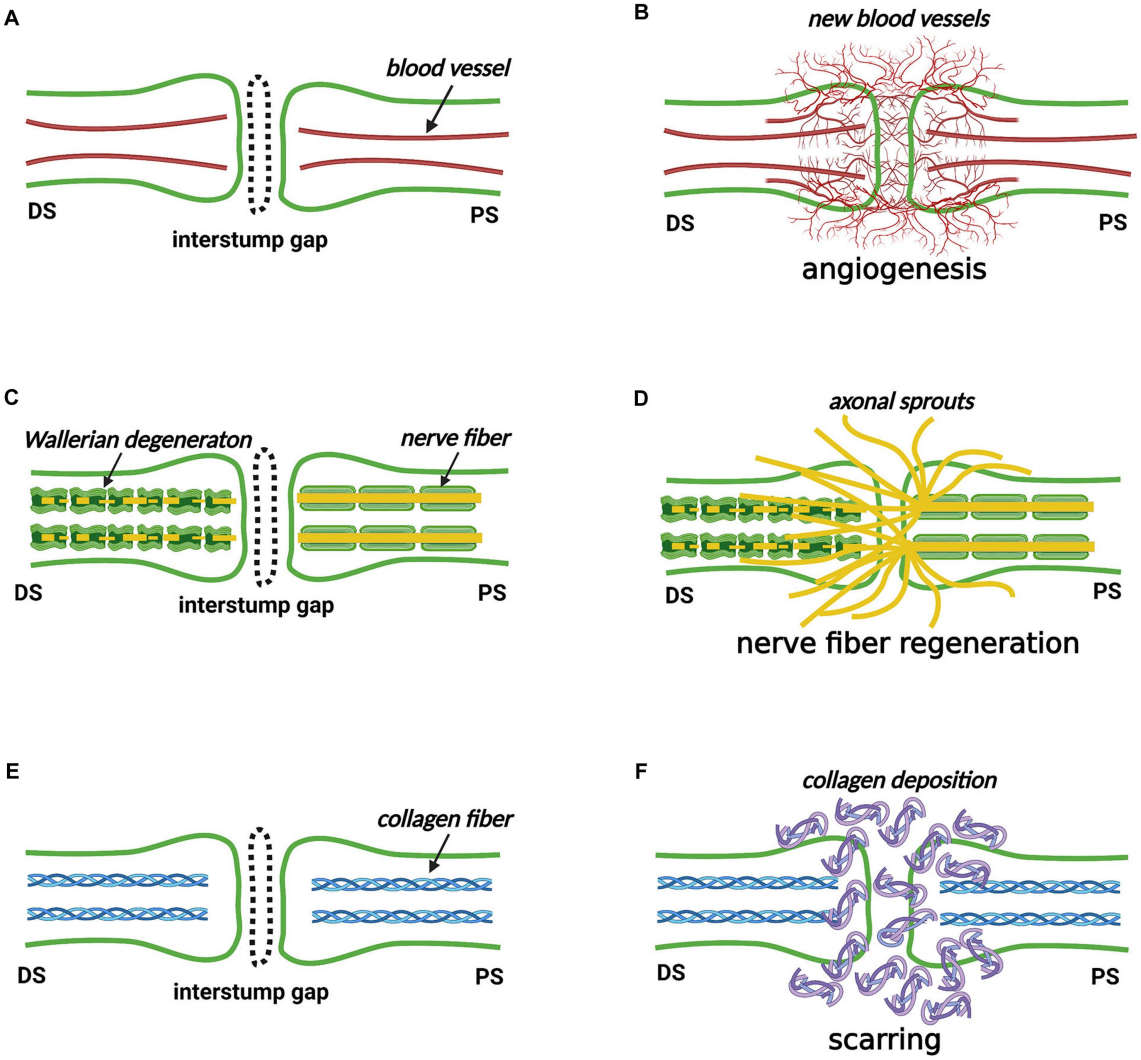
The nerve trunk healing process at the nerve transection site (Created with BioRender.com). **(A,B)** Angiogenesis occurs in the proximal stump, distal stump, interstump gap, and surrounding tissue bed. **(C,D)** Nerve fibers own a robust ability to regenerate from the proximal stump. Some nerve fibers cross the interstump gap and subsequently enter the distal stump where Wallerian degeneration occurs, but others enter the surrounding tissue bed. **(E,F)** Scarring by depositing collagen fibers occurs to reconstitute the interstump gap. DS, distal stump; PS, proximal stump.

Inspired by the article “Healing of Nerves” by Dr. Hanno Millesi and the classic pathological theory of skin wound healing as well as the monograph “The Healing of Nerves” by Sir Charles Ballance and Sir James Purves-Stewart, we recommend that the phrase “nerve trunk healing” might be used to represent the reconstitution of the interstump gap by the “new structure,” which reconnects the two separated nerve stumps (35, 36). Teleologically, we speculate that three basic requirements must be met for a transected nerve trunk to heal its architecture as similar as possible to its original state: (i) the “new structure” must contain blood vessels supplying oxygen and nutrients, which is accomplished by angiogenesis; (ii) the “new structure” must involve nerve fibers transmitting electrical stimulus, which is accomplished by nerve fiber regeneration; (iii) the “new structure” must restore the connective tissue layers maintaining the tensile strength and the gliding mobility, which is accomplished by scarring. Accordingly, we infer that angiogenesis, nerve fiber regeneration, and scarring must occur at the nerve transection site (see Sections 4–6) (Figure 4).

The concept of nerve trunk healing interprets the pathological and physiological processes occurring in the interstump gap after nerve transection injury at the organ level. We separate the nerve trunk healing process, at the tissue level, into three concurrent responses, namely, angiogenesis, nerve fiber regeneration, and scarring. This thinking pattern may provide new insight for understanding what is happening at the nerve transection sites.

In pathology, the concept of “healing” and “repair” are used interchangeably (37). In this sense, the phrase “nerve trunk healing” and “nerve trunk repair” may be used synonymously. However, considering the potential miscomprehension with the phrase “surgical repair,” we recommend the phrase “nerve trunk healing.”

### 3.3. A conceptual concern about “regeneration”

A question probably will emerge about whether the phrase “nerve trunk regeneration” is more appropriate than the phrase “nerve trunk healing” at the nerve transection site. We have found an inapparent controversy about the term “regeneration,” which has been used widely in references concerning peripheral nerve injury.

Based on the general pathological theory, regeneration is accomplished by the proliferation of residual undamaged cells, and the outcome of regeneration is restoring the “original” or “normal” architecture of damaged parts (38). To our knowledge, “new structures” forming in interstump gaps (see Section 3.1) have not been anatomically identical to the original nerve trunks. In this sense, at the nerve transection site, nerve trunks do not pathologically regenerate either at the organ level or at the tissue level.

Outside the field of medicine, “regeneration” is an ambiguous term with multiple meanings in both regenerative biology and social science (39, 40). Properly speaking, nowadays, the field of “peripheral nerve regeneration” has been innovatively studied by neurobiologists and tissue engineers (3, 41). The exact meaning of the phrase “peripheral nerve regeneration,” in most situations, is “regeneration of nerve fibers within peripheral nerves,” “nerve fiber regeneration” or “axonal regeneration and remyelination,” rather than “nerve trunk regeneration.”

In pathology, the concept of “repair (or healing)” consists of “regeneration” and “scarring” (see Section 5.1). If the phrase “nerve trunk regeneration” had to be used, it would be applied in the situation of nerve crush injury in which the connective tissues and blood vessels reserve the continuity.

### 3.4. The neuroma formation at nerve transection sites

**Figure 5:**
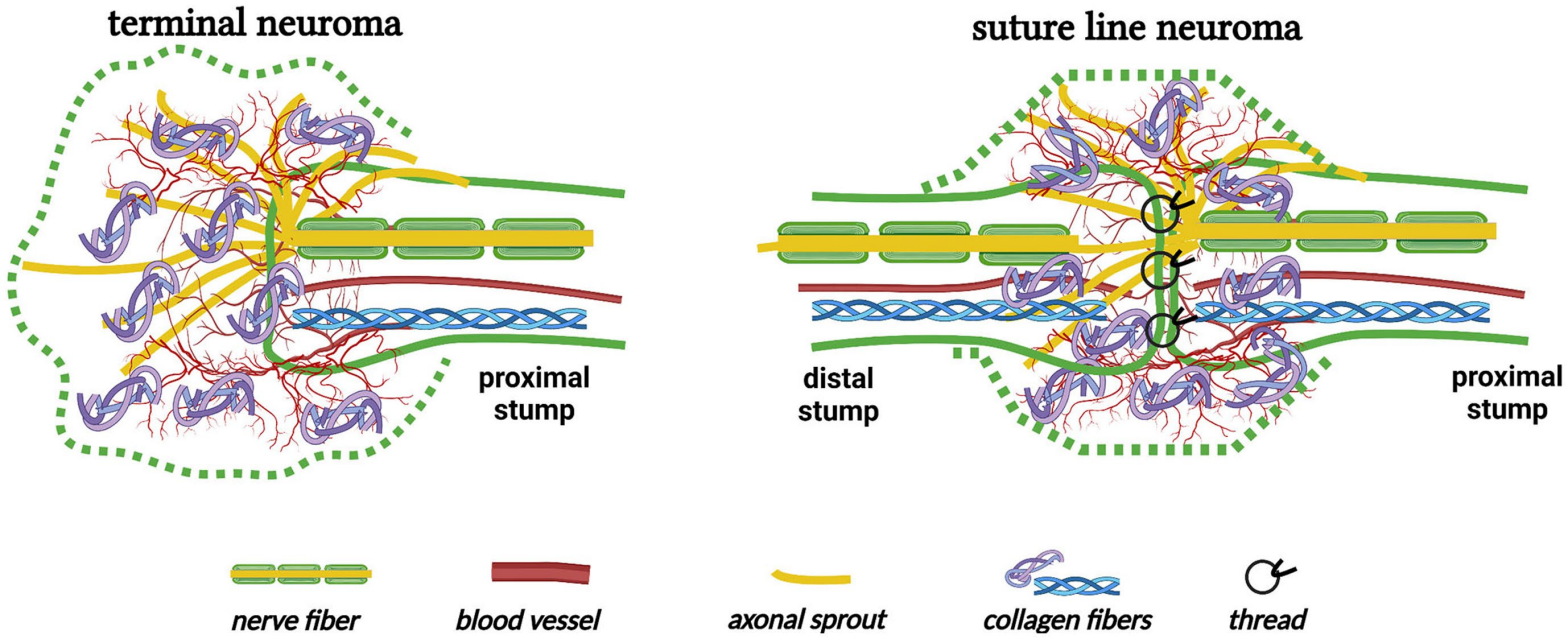
The formation of terminal neuroma and suture line neuroma (Created with BioRender.com). A terminal neuroma revealing the chaos of regenerating nerve fibers, blood vessels, and collagen fibers grows at the end of the proximal stump when there is no distal stump. Similarly, a suture line neuroma forms at the nerve transection site even after the interstump gap is surgically repaired.

Another change in nerve trunks after traumatic injury is neuroma formation. Simply, neuromas are divided into “terminal neuromas” and “neuromas-in-continuity” (42). The terminal neuroma, which reveals the chaos of regenerating axons, Schwann cells, blood vessels, fibroblasts, and connective tissues, inevitably grows at the end of the proximal stump when there is no distal stump or the interstump gap is too long (Figure 5) (43, 44) “Neuroma after nerve repair” is one subtype of neuroma-in-continuity (45, 46). Alternative phrases, such as “suture line neuroma” (Figure 5) (30, 31), “neuroma scar” (32), or simply “neuroma” (28, 29, 47, 48), refer to the masses or swellings at the surgical repair sites in animal experiments (also see Section 3.1). These investigations indicate that even surgically repaired interstump gaps can not avoid the formation of suture line neuromas.

### 3.5. The relationship between nerve trunk healing and neuroma formation

Regardless of nomenclature and terminology, in animal experiments, the “new structure” we mentioned in Section 3.1 is likely identical to the nature of the “neuroma” in Section 3.4. A question may arise about how to interpret the intrinsic relationship between nerve trunk healing and neuroma formation. The two processes may seem unrelated, even opposite, but counterintuitively, they are probably two sides of the same coin. The reasons are as follows. First, they are both initiated by the nerve transection injury. Second, both the “new structure” within the interstump gap and the neuroma consist of blood vessels, regenerating axons, and scar tissues. It indicates that new blood vessel formation, scar tissue deposition, and nerve fiber regeneration must occur at the tissue level. Third, above all, cellular responses and molecular mediators underlying the two processes may be indistinguishable and indistinctive, because cells and molecules that emerge at the nerve transection site (such as hemocytes, macrophages, fibroblasts, endothelial cells, Schwann cells, cytokines, growth factors, and extracellular matrix molecules) are identical and probably under similar regulations.

Collectively, it is rational to consider neuromas as the result of the process of nerve trunk healing after nerve traumatic injury. Furthermore, the formation of neuromas and the healing of nerve trunks are the same neurobiological event. In this sense, terminal neuromas are the results of the nerve trunk healing process at the end of the proximal stump; and suture line neuromas are consequences of this process after neurorrhaphy at the nerve transection sites. Therefore, the knowledge or clues from the field of “peripheral nerve injury,” “peripheral nerve regeneration,” and “neuroma formation” should not be separated.

### 3.6. Clinical considerations

Clinically, nerve transection injuries generated by daily trauma in humans may not be as uniform as those produced in laboratory animals. Surgeons develop various techniques to close interstump gaps of different sizes, such as end-to-end neurorrhaphy, nerve transferring, and nerve grafting (49). Surgery provides the only opportunity for a transected nerve to restore the innervation of the target organs in clinical practice (43), but what is noteworthy is that the fundamental role of surgical intervention is to juxtapose two ends of stumps and shorten the interstump gap mechanically. Even though a transected nerve trunk is microsurgically repaired, an interstump gap of minimum length remains at the surgical repair site (36, 50). Closing the interstump gap histologically is accomplished only by the nerve trunk healing process. Comprehending the nerve trunk healing process after surgical intervention is a biological issue whose secrets are not only needles, threads, conduits, or microscopes (51).

Although most neuromas are asymptomatic, in the practice of hand surgery, about one in 15 patients will suffer from a painful neuroma after digital amputation (52). In the practice of neurology, nerve biopsy is one kind of invasive operation related to terminal neuromas (53). Physical therapy, oral medications, steroid injections, ablation, and surgical management are applied in the treatment of painful neuromas, but there is no single strategy that completely and consistently eliminates neuromas, and the best treatment for a neuroma is probably to prevent its formation (45). According to the discussion in Section 3.5, understanding the nature of the nerve trunk healing process may contribute to exploring new strategies for the prevention or treatment of neuromas, especially terminal neuromas.

## 4. Behaviors of nerve fibers after transection injuries

From the proximal stump, nerve fibers regenerate. The average number of axonal sprouts from their parent axon is five (3). Moreover, every axonal sprout owns one growth cone at its end (45). Each “regeneration unit” originates from a single myelinated axon (30, 54). The original large fascicles in the proximal stump disappear and are replaced by “minifascicles” (55). This process is named “compartmentation” (55). It is logical that before an axonal sprout reinnervates into the target organ, it must cross the interstump gap and subsequently enter the distal stump. Regenerating units and minifascicles are observed not only in the proximal stump but also in the interstump gap and the distal stump (30, 31, 56). Remarkably, in the distal stump, the total number of regenerated nerve fibers increases compared with it before the nerve transection injury (30). This is consistent with numerous axonal sprouts growing from the proximal stump. This observation indicates that nerve fibers own a robust ability to cross the interstump gap.

Within the distal stump, nerve fibers degenerate (Wallerian degeneration), but connective tissues survive (57–60). In the extrafascicular area of the distal stump, there are numerous regenerating units, which reveal loss of axonal sprouts (30, 31); in the intrafascicular area, axons fail to regrow through their original pathway (61). Therefore, the functional recovery of nerve transection injury is never complete (62).

## 5. Responses of connective tissues at nerve transection sites

### 5.1. The nerve trunk healing process must contain scarring

On the general pathological principle, the tissue-repair process involves two biological reactions: regeneration by the proliferation of cells and scarring (scar formation, fibrosis) by depositing collagen fibers from fibroblasts (38). This theory is best compatible with the skin wound healing process by the second intention, in which the epidermis, the basement membrane, and the dermis are all destroyed following a significant tissue defect (63). After the transection injury of a peripheral nerve trunk, the normal wound healing cascade initiates around the surrounding tissue bed (64). In the interstump gap where Schwann cell basal laminae and connective tissue layers lose their continuity, the nerve trunk healing process may not differ from lacerated skin but is probably more complicated. Thus, we suggest that the reconstitution of the interstump gap on the nerve trunk must include scarring. In other words, appropriate scarring may be favorable for the normal nerve trunk healing process, but excessive scarring probably contributes to adverse nerve trunk adhesion.

### 5.2. The hypothesis that the scar impedes peripheral axon regeneration

In the central nervous system, the so-called “glial scar” forms at the site of tissue damage, which is the main barrier for central axons to regenerate (59, 65). The glial scar is mainly composed of reactive astrocytes and proteoglycans (65). Properly speaking, the glial scar is not the so-called “scar tissue” that consists of fibroblasts and collagens. The concept that central axons can not cross glial scars is widely accepted (60, 65).

Nevertheless, in the peripheral nervous system, it is controversial whether scarring at the surgical repair site is a major contributing factor for incomplete neural function recovery. Based on Milles’s works, Lundborg stated that scarring at the distal repair site of a nerve graft might prohibit axonal sprouts, but this situation is very uncommon if the tension of the repair site is avoided (5). Similarly, scarring at the surgical repair site is not included in the eight factors influencing neural function recovery after surgical manipulation (43). In contrast, several investigations support the hypothesis that the scarring at the surgical repair site forms a mechanical barrier that impedes axonal regeneration and subsequently impairs neural function recovery (28, 29, 64, 66–69). Meanwhile, this barrier presumably contributes to a “neuroma scar” consisting of misdirected axonal sprouts (32). Moreover, this barrier probably functions as an origin of the ectopic neural discharge of injury-induced pain (70, 71).

### 5.3. Negative correlation between scarring and axonal regeneration

One strategy for improving the outcome of the nerve trunk healing process after transection injury is permitting more nerve fibers to regrow into the distal stump by preventing scar formation at the surgical repair site using pharmacological agents or anti-adhesion equipment (72–74). The underlying logic of this strategy is the hypothesis that scarring might impede axonal regeneration at the nerve transection site (see Section 5.2). Atkins and colleagues first reported a negative correlation between the level of scarring and two indicators of neural function recovery (the axon counting number and the electrophysiological parameter) at the surgical repair site following nerve transection injury in 6 weeks (68). They seminally used two types of transgenic mice: a more-scarring model and a less-scarring model.

**Figure 6:**
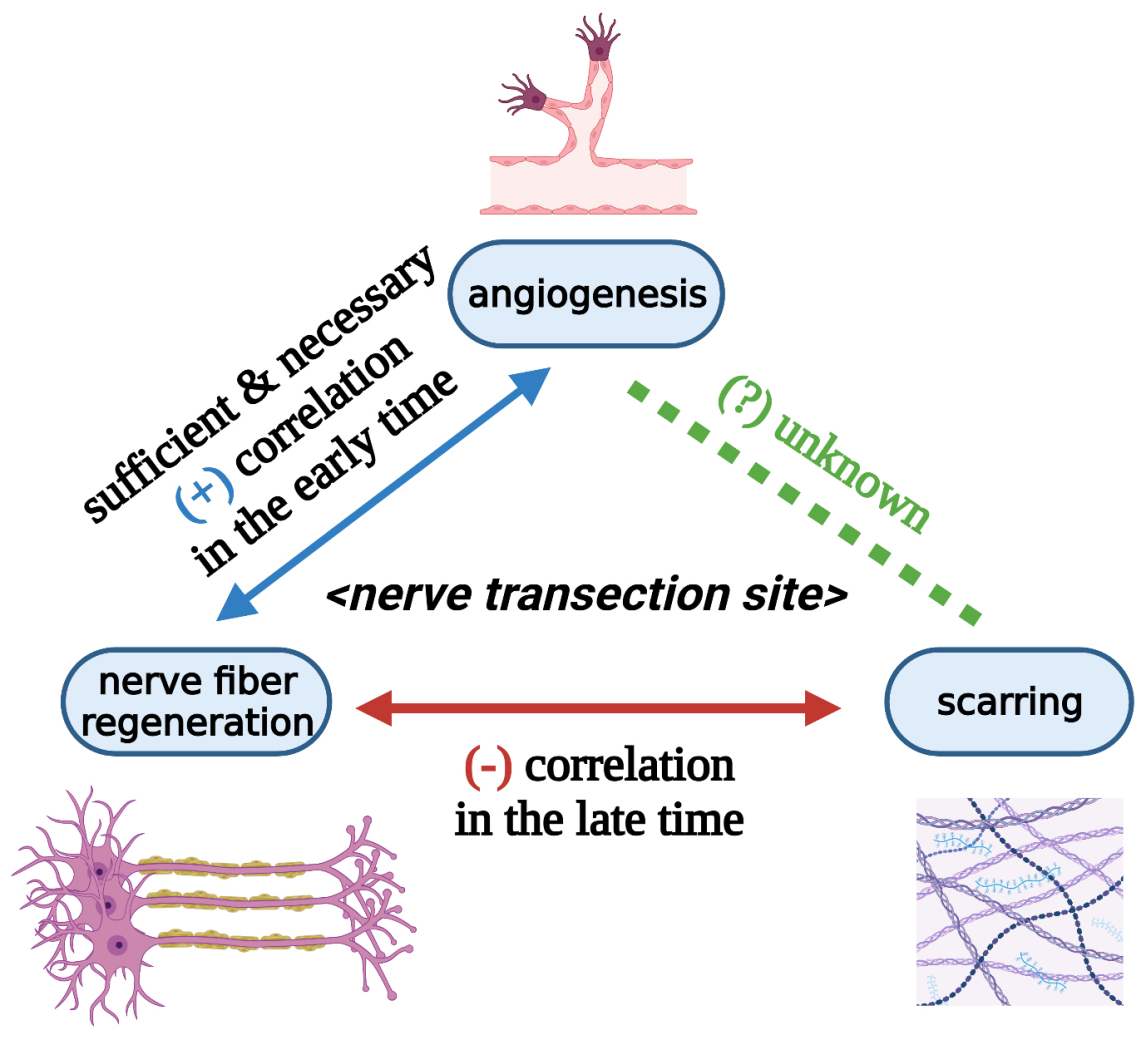
Relationships among angiogenesis, nerve fiber regeneration, and scarring at peripheral nerve transection sites at the tissue level (Created with BioRender.com). Angiogenesis at the nerve transection site is sufficient and necessary for nerve fiber regeneration in the early time. Angiogenesis and nerve fiber regeneration show a positive correlation in the early time. Scarring and nerve fiber regeneration reveal a negative correlation in the late time. The relationship between angiogenesis and scarring during nerve trunk healing is still unknown.

In this section, we pay close attention to locally-administrated agents that reveal pharmacological effects of scar inhibition and axonal regeneration stimulation at the surgical repair site in nerve transection models (Table 1) (29, 75–77). Additionally, the intragastrical administration of FK506 (4 mg/kg per day for 2, 4, and 6 weeks) contributes to improved nerve fiber regeneration, accelerated neural function recovery, and decreased scarring in the same rat model (78). These studies strongly indicate that a negative correlation exists between scarring and axonal regeneration as well as between scarring and neural function restoration in the late phase (Figure 6) (29, 75–78). However, it is probably reluctant to demonstrate a cause-and-effect relationship between scarring and nerve fiber regeneration.

**Table 1:** Locally-administrated agents revealing pharmacological effects of scar inhibition and axonal regeneration stimulation at surgical repair sites in sciatic nerve transection models of animals.

Agent	Explanation	Dosage	Animal	Observation time	Pharmacological effects
IL-10 (29)	Anti-inflammatory cytokine	125 ng in 100 μl	C57-Black-6 mouse	6 weeks	To decrease scarring
					To improve electrophysiological restoration
M6P (75)	TGFβ inhibitor	600 mM × 100 μl	C57-Black-6 mouse	6 weeks 12 weeks	To reduce scarring
					To enhance electrophysiological recovery
					To enhance behavior recovery
FK506 (76)	Immunosuppressive drug	10 ng/mL × 15 μl	European rabbit	12 weeks	To prevent scarring
					To enhance nerve fiber regeneration
HA (76)	Mucopolysaccharide (a natural component of the extracellular matrix)	4 mg in 0.5 ml	European rabbit	12 weeks	To prevent scarring
					To enhance nerve fiber regeneration
HA-CMC (77)	Solution of mucopolysaccharide and biocompatible polysaccharide	1 ml	Sprague–Dawley rat	12 weeks	To reduce scarring and adhesion
					To enhance the organization of regrowing axons

TGFβ, transforming growth factor beta; IL-10, interleukin-10; M6P, mannose-6-phosphate; FK506, tacrolimus; HA, hyaluronic acid; CMC, carboxymethylcellulose.

## 6. Reactions of blood vessels after nerve transection injuries

### 6.1. Clot formation and inflammatory reaction after nerve transection injuries

Experimental studies *in vivo* and clinical experiences have revealed that an open wound of living vascularized organisms must bleed. Complete transected nerve trunks are no exceptions and must result in vascular injury at the injury site. For transected nerves, the highest priority is to stop bleeding by hemostasis. The clot physically plugs the interstump gap and the surrounding tissue bed to minimize blood loss. If the tension of nerve stumps is completely avoided, the fibrin clot may be sufficient to maintain the coaptation between nerve stumps (36).

Cell and tissue damage by traumatic injuries inevitably occur at the transected nerves. Since inflammation is a response of vascularized tissues and organs bringing leukocytes and molecules to the sites of injury to eliminate the hostile agent (38), the transected nerve trunk must undergo the inflammatory response. In the standard nerve transection model and under the sterile condition, the injury site may experience acute inflammation, which induces vascular reactions (vasodilation and angiogenesis) as well as sets motion to the nerve trunk healing process and finally resolves.

### 6.2. Angiogenesis at nerve injury sites

Angiogenesis is under special attention by pathologists, oncologists, and plastic surgeons. In the mindset of pathologists, angiogenesis (or neovascularization) is one step of the tissue-repair process, which is set in motion by inflammation (38). In the thinking pattern of oncologists who support the balance hypothesis for angiogenesis (see Section 7.1), the metastasis of tumor cells and the metastasis growth to a clinically detectable size depend on angiogenesis (79). In the conventional thinking of plastic surgeons, vascularization of the tissue-engineered products or the tissue grafts by angiogenesis, vasculogenesis, and inosculation is one major issue (80–82). To our knowledge, not many investigations concerning angiogenesis at nerve injury sites exist. Rare are studies of angiogenesis at the transected nerve suture lines. Angiogenesis is directly observed in the proximal stump, distal stump, interstump gap, and surrounding tissue bed (Figure 4) (9, 19, 21, 25, 83–86). Here, we propose that mechanisms of angiogenesis at the nerve injury site may not be different from those during the inflammatory response, tumorigenesis, and tissue grafting.

#### 6.2.1. Angiogenesis in the Interstump gap of nerve transection sites

The limitation of oxygen diffusion through normal tissues is 100–200 μm (87). Generally, cells beyond 150 μm from blood vessels fail to survive (88, 89). The 200 μm may be the minimum length of the interstump gap, which spontaneously forms within rodent nerves following transection injury (26, 27). This information suggests that the interstump gap must be in decreased oxygen levels (or hypoxia) because there are no blood vessels in the interstump gap at the initial time. Cattin and colleagues proved that the interstump gap of transected rat sciatic nerve is under a hypoxic microenvironment in the initial 2 days, containing few blood vessels (25). In 3 days, the interstump gap of the rat sciatic nerve significantly vascularizes by a dramatic influx of blood vessels, indicating angiogenesis occurs; in mice, this process takes 5 days (25).

#### 6.2.2. Angiogenesis of the interstump gap in the nerve regeneration chamber

Podhajsky and Myers investigated the angiogenesis in a 10-mm gap within the silicone chamber of the rat sciatic nerve transection injury (86). Although the vascular growth occurs from both the proximal and distal stumps, the traveling wave of angiogenesis in the chamber appears in the proximal-distal direction (86). This direction is consistent with the nerve graft revascularization reported in other research (90, 91).

#### 6.2.3. Angiogenesis within the proximal and distal stumps

In the proximal stump of the transected nerve trunk, angiogenesis is observed at least 7 days after nerve injury (19). Similarly, in the distal stump of the transected nerve trunk, prominent angiogenesis is observed 14 days after nerve injury (21). In both stumps, angiogenesis is most apparent in the epineurium and perineurium (19, 21).

Podhajsky and Myers studied the vascular response (the spatial and temporal change of the total endothelial surface area within the intrafascicular area) at the rat sciatic nerve crush injury site and its distal stump (10-mm length) from 1 week to 9 weeks (92). In the early phase, peaked in the first week, the vessel size increases but not the vessel number, which may indicate vasodilation; in the second phase, peaked in the sixth week, the vessel number and density increase, which may indicate angiogenesis (92).

### 6.3. Angiogenesis is sufficient and necessary for nerve fiber regeneration

Previously, Caillaud and colleagues reviewed Wallerian degeneration, axonal regeneration, and revascularization in the distal stump after nerve traumatic injury (93). In this section, we mainly focus on the relationship between angiogenesis and nerve fiber regeneration in the interstump gap of the nerve transection site.

Hobson and colleagues provided direct evidence for the structural relationship between angiogenesis and nerve fiber regeneration within the 10-mm interstump gap of rat sciatic nerve fibronectin conduit as early as the 10th day (85). Stimulated angiogenesis by local administration of exogenous recombinant human vascular endothelial growth factor (rhVEGF) accompanies the enhancement of nerve fiber regeneration within the 10-mm interstump gap of rat sciatic nerve silicon conduit on the 10th day (94). These lines of evidence likely suggest a positive correlation between angiogenesis and nerve fiber regeneration in the interstump gap in the early time (Figure 6).

Schwann cells, accompanied by growing axons, migrate into the aberrant angiogenesis areas around rhVEGF-loaded heparin beads in distal stumps, gaps, and proximal stumps of rat sciatic nerve transection model without surgical anastomosis on the 6th day (25). Inhibited angiogenesis by local administration of exogenous VEGFA antibody is parallel with impairment of nerve fiber regeneration within the interstump gap of mouse inferior alveolar nerve transection model without surgical anastomosis on the 7th day (95). Inhibition of angiogenesis by oral administration of cabozantinib (inhibitor of VEGF receptor 2) blocks the entry of Schwann cells and axons into the interstump gap in sciatic nerve transection model of PLP-EGFP transgenic mice (mice of which Schwann cells specifically express the enhanced green fluorescent protein (EGFP)) on the 5th day (25). These phenomena indicate the angiogenesis induced by VEGF-VEGFR signaling (see Section 7.2) is sufficient and necessary for nerve fiber regeneration at the nerve transection site in the early time (Figure 6). Furthermore, angiogenesis is likely one of the causes of nerve fiber regeneration at the nerve transection site.

After 6 months, the transverse structure of the distal stump of *Vegfa^fl/fl^ Tie2-Cre* mice (mice lacking *Vegfa* in hematopoietic cells and endothelial cells, and its anti-angiogenic effect is dependent on cells derived from hematopoietic stem cells) is not visibly different from the *Vegfa^fl/fl^* control mice, except that the whole distal stump of the former is visibly smaller than the latter (25). This evidence indicates that a normal angiogenic process by VEGF-VEGFR signaling (see Section 7.2) in the interstump gap is necessary for effective nerve fiber regeneration in the distal stump in the late phase.

### 6.4. Inferring the relationship between angiogenesis and scarring

Both angiogenesis and scarring are indispensable for mammalian survival (96, 97). In Section 5.3, we infer a negative correlation between scarring and nerve fiber regeneration at the nerve transection site in a late state (at least 6 weeks). In Section 6.3, we infer a positive correlation between angiogenesis and nerve fiber regeneration at the nerve transection site in an early phase (about 1 week). Further, the question arises about how angiogenesis and scarring interact at the nerve transection site if this crosstalk exists. To our knowledge, we have not found any literature to address this question (Figure 6). Spatially, angiogenesis and scarring occur at the same nerve transection site. Temporally, angiogenesis accomplishes as early as 48 hours at the nerve transection site (9); scarring initiates with traumatic injury and proceeds for an extended period (weeks to months) (38, 98, 99). Hence, considering a simple linear correlation between angiogenesis and scarring at nerve transection sites is probably unreasonable.

If angiogenesis does not occur at the nerve transection site, there is likely no granulation tissue or mature scar in the interstump gap and surrounding tissue bed. Subsequently, the nerve trunk healing process may fail. Hence, we speculate that angiogenesis may be necessary for scarring in nerve transection injury.

## 7. The knowledge about angiogenesis from tumor therapy

### 7.1. The balance hypothesis for angiogenesis

Dr. Judah Folkman first hypothesized that anti-angiogenesis might be a potential therapeutic strategy for curing human cancers 52 years ago (100, 101). According to the balance hypothesis for the angiogenic switch, the relative balance of activators and inhibitors can keep the switch of angiogenesis on or off (102). Although angiogenesis does not cause carcinomas or sarcomas, it facilitates tumor progression and metastasis (97). Hence, the underlying logic of the anti-angiogenic therapy for solid tumor treatments is that angiogenesis is necessary for metastatic tumor cells to escape from a primary tumor as well as for a metastatic tumor to grow into a clinically inspected volume (79).

### 7.2. VEGF-VEGFR signaling

**Figure 7:**
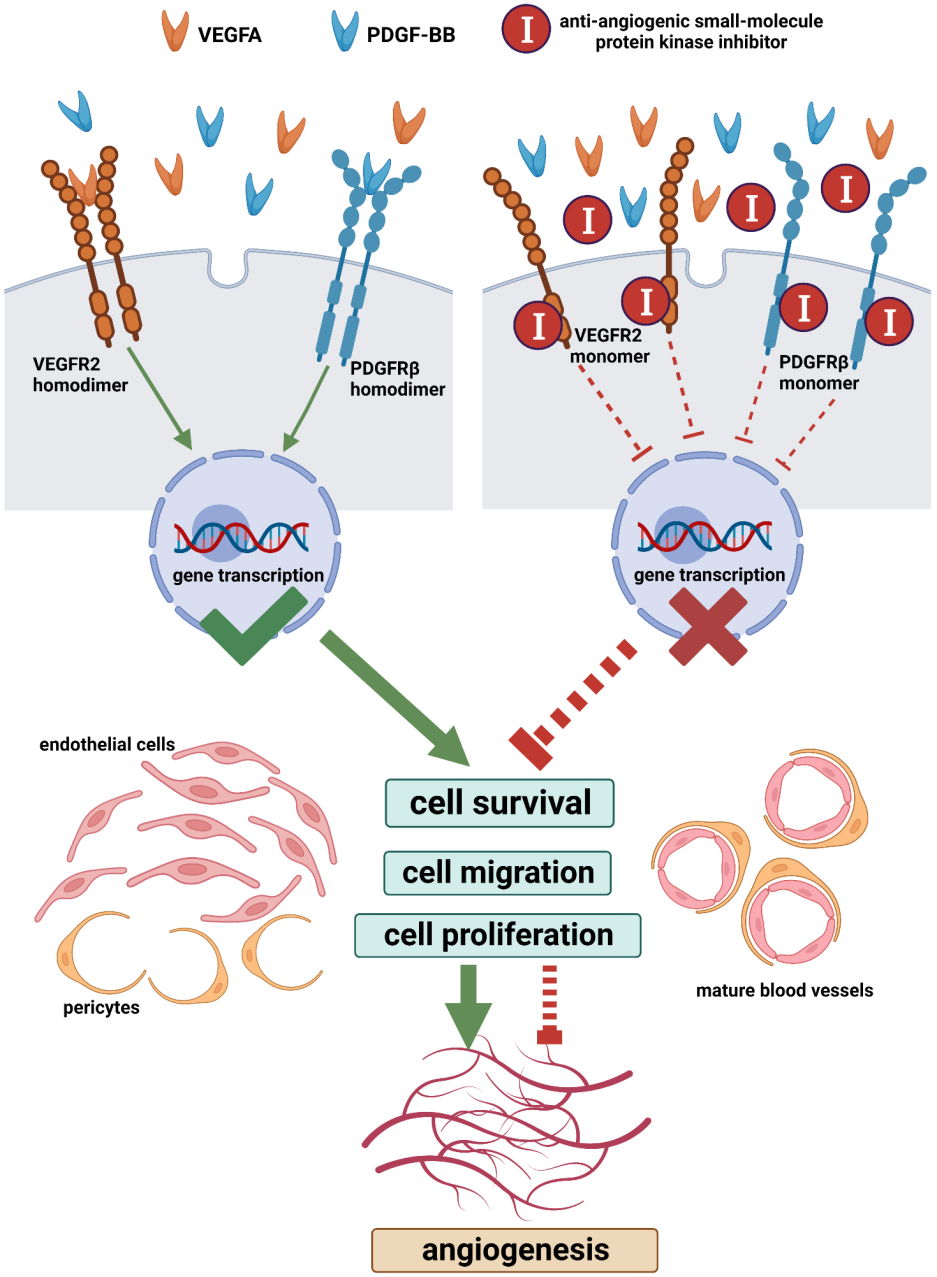
The main machinery of the angiogenic signaling pathway and its blocking with anti-angiogenic small-molecule protein kinase inhibitors (Created with BioRender.com). When a VEGFA dimer combines with two VEGFR2 monomers on the cell membrane, these VEGFR2 monomers dimerize and auto-phosphorylate, which initiates downstream signaling cascades leading to cell survival, migration, and proliferation. Similarly, following the combination of a PDGF-BB dimer and two PDGFRβ monomers, these PDGFRβ monomers dimerize and auto-phosphorylate. The VEGFA-VEGFR2 pathway is implicated in every aspect of angiogenesis. PDGFRβ regulates the recruitment of pericytes and the (PDGF-BB)-PDGFRβ pathway is essential for vascular maturation. The simultaneous blocking of the VEGFR and PDGFR with multi-target small-molecule protein kinase inhibitors is an effective anti-angiogenic strategy. VEGFA, vascular endothelial growth factor a; PDGF-BB, homodimer of platelet-derived growth factor b chain; VEGFR2, vascular endothelial growth factor receptor 2; PDGFRβ, platelet-derived growth factor receptor beta.

The mammalian vascular endothelial growth factor and its receptor (VEGF/VEGFR) family members consist of five dimeric VEGFs (VEGFA, VEGFB, VEGFC, VEGFD, and placenta growth factor (PLGF)), three monomeric receptors (VEGFR1, VEGFR2, and VEGFR3), and co-receptors [neuropilin family (NRP1 and NRP2) and heparan sulfate proteoglycans (HSPGs)] (103). In common situations, VEGF refers to VEGFA (100, 104). VEGFs are extensively expressed not only in the vasculature but also in the central nervous system, kidney, lung, and liver (105, 106). In adults, VEGFR1 (also FLT1) and VEGFR2 (also FLK1/KDR) are found mainly in blood vascular endothelial cells while VEGFR3 (also FLT4) is predominantly expressed in lymphatic endothelial cells where it regulates lymphangiogenesis (87, 107). VEGFR1 is also localized in monocytes, macrophages, vascular smooth muscle cells, neuronal cells, and tumor cells; VEGFR3 is also found in neuronal progenitors, macrophages, and osteoblasts (103, 108, 109). VEGFR2 is the chief mediator for the roles of VEGFA in both slow responses through gene regulation (such as endothelial cell survival, migration, and proliferation) and quick responses (such as vessel permeabilization) (Figure 7) (100, 108–110). The VEGFA-VEGFR2 signaling pathway is implicated in every aspect of physiological and pathological angiogenesis (109). When a dimeric VEGFA molecule combines with two monomeric VEGFR2 molecules on the endothelial cell membrane, these VEGFR2 molecules dimerize and auto-phosphorylate, subsequently, initiating numerous downstream signaling cascades (Figure 7) (109). Hence, strategies to block the VEGF-VEGFR signaling pathway for tumor therapy have been investigated (104, 111).

### 7.3. PDGF-PDGFR signaling

The mammalian platelet-derived growth factor and its receptor (PDGF/PDGFR) family members are composed of five dimeric PDGFs (PDGF-AA, PDGF-BB, PDGF-CC, PDGF-DD, and PDGF-AB) and two monomeric receptors (PDGFRα and PDGFRβ) (112, 113). Following the combination of a dimeric PDGF molecule and two monomeric PDGFR molecules, these PDGFR molecules dimerize and auto-phosphorylate (Figure 7) (114). PDGFs are expressed by different cell types including endothelial cells, vascular smooth muscle cells, Schwann cells, fibroblasts, macrophages, and so on (114). During mammalian development, *in vivo*, PDGF-AA and PDGF-CC interact with PDGFRα homodimers while PDGF-BB interacts with PDGFRβ homodimers (112). In the developing vasculature, PDGF-B is synthesized by endothelial cells and PDGFRβ is expressed in mural cells (pericytes and vascular smooth muscle cells) (115). During angiogenesis, PDGFRβ regulates the recruitment of pericytes and the (PDGF-BB)-PDGFRβ signaling pathway is essential for vascular maturation (Figure 7) (116).

### 7.4. Anti-angiogenic small-molecule protein kinase inhibitors to multiple targets

The prerequisite for VEGFs and PDGFs to reveal their biological effects is that the target cells express VEGFRs and PDGFRs on their plasma membranes (Figure 7). The nature of VEGFRs and PDGFRs are protein kinases (PKs) (117). Nowadays, the United States Food and Drug Administration (U.S. FDA) has approved 73 small-molecule protein kinase inhibitors (PKIs), summarized by Roskoski (118, 119). These small molecules have been selected from a large number of chemical compounds during time-spending laboratory investigations and clinical trials (120). Since the concurrent blockage of the VEGF-VEGFR and PDGF-PDGFR signaling pathways is an attractive anti-angiogenic strategy, we searched for anti-angiogenic small molecules that simultaneously inhibit VEGFRs and PDGFRs among these 73 drugs and subsequently selected nine candidates, namely, sorafenib, sunitinib, pazopanib, axitinib, ponatinib, regorafenib, nintedanib, lenvatinib, and tivozanib (Figure 7 and Table 2) (110, 118, 119, 121–138). Additionally, it is notable that these nine anti-angiogenic small molecules are multi-target drugs.

**Table 2:** U.S. FDA-approved small-molecule PKIs simultaneously inhibiting VEGFR and PDGFR.

Approved year	Drug	Medicine name	Targeting VEGFR	Targeting PDGFR	Other targets
2005	Sorafenib (121)	NEXAVAR^®^ Tablet	VEGFR1/2/3	PDFGRβ	KIT, FLT3, RET, c-Raf, b-Raf
2006	Sunitinib malate (122)	SUTENT^®^ Capsule	VEGFR1/2/3	PDGFRα/β	KIT, FLT3, RET, CSF1R
2009	Pazopanib (123)	VOTRIENT^®^ Tablet	VEGFR1/2/3	PDGFRα/β	FGFR1/3, KIT, ITK, LCK, CSF1R
2012	Axitinib (124–127)	INLYTA^®^ Tablet	VEGFR1/2/3	PDGFRα/β	KIT
2012	Ponatinib (128)	ICLUSIG^®^ Tablet	VEGFR	PDGFR	FGFR, KIT, FLT3, RET, Eph, Src, Tie2, BCR-ABL
2012	Regorafenib (129)	STIVARGA^®^ Tablet	VEGFR1/2/3	PDGFRα/β	FGFR1/2, KIT, RET, CSF1R, DDR2, TrkA, Eph, Tie2, c-Raf, b-Raf
2014	Nintedanib (130)	OFEV^®^ Capsule	VEGFR1/2/3	PDFGRα/β	FGFR1/2/3, FLT3, CSF1R, LCK, Lyn, Src
2015	Lenvatinib (131)	LENVIMA^®^ Capsule	VEGFR1/2/3	PDGFRα	FGFR1/2/3/4, KIT, RET
2021	Tivozanib (132–134)	FOTIVDA^®^ Capsule	VEGFR1/2/3	PDGFRβ	KIT

U.S. FDA, United States Food and Drug Administration; PKI, protein kinase inhibitor; VEGFR, vascular endothelial growth factor receptor; PDGFR, platelet-derived growth factor receptor; PDGFRα, platelet-derived growth factor receptor alfa; PDGFRβ, platelet-derived growth factor receptor beta; FGFR, fibroblast growth factor receptor; KIT, stem cell factor receptor; FLT3, Fms-like tyrosine kinase 3; RET, glial-derived neurotrophic factor receptor; CSF1R, colony-stimulating factor 1 receptor; ITK, interleukin-2-inducible T-cell kinase; LCK, lymphocyte-specific protein tyrosine kinase; DDR2, discoidin domain receptor 2; BCR-ABL, the protein translated from the *BCR-ABL* gene which is located in the Philadelphia chromosome.

## 8. A new hypothesis and ways to test

### 8.1. The hypothesis that anti-angiogenesis suppresses neuromas

According to the discussion in Sections 4–6, the essential prerequisite for nerve trunk healing is angiogenesis at the nerve transection site. If the angiogenesis is inhibited, nerve fiber regeneration and scarring may be unaccomplished. Subsequently, the nerve trunk healing process may frustrate. Inversely, if the angiogenesis is stimulated, nerve fiber regeneration and scarring, at least, may not be impaired. If inhibition of angiogenesis at the nerve transection site contributes to the impairment of nerve trunk healing, based on Section 3, the anti-angiogenic strategy may be beneficial for the prevention or treatment of neuromas, particularly terminal neuromas.

The capacity for transected nerve fibers to regenerate is robust (see Section 4). Numerous axonal sprouts rise from the proximal stump, cross the interstump gap, and enter the intrafascicular area of the distal stump; meanwhile, they also misdirect to the extrafascicular area in both the distal and proximal stumps (19, 30, 54, 55). This misdirection of nerve fibers to the extrafascicular area leads to the loss of new nerve fibers in the intrafascicular area of the distal stumps and may contribute to the formation of suture line neuromas at the surgical repair sites. The extrafascicular area of a nerve trunk is abundant for connective tissue and blood vessels, where angiogenesis and scarring mainly occur after nerve transection injury (19, 21, 66). Accordingly, the question arises about whether the inhibition of extrafascicular angiogenesis contributes to avoiding nerve fiber loss and scarring at the nerve transection site as well as preventing suture line neuromas.

Here, we hypothesize that anti-angiogenic therapy can suppress both terminal neuromas and suture line neuromas. The underlying mechanism is that angiogenic inhibition probably contributes to the failure of nerve fiber regeneration and scarring.

### 8.2. Feasible routes to verify our hypothesis

In the future, we suggest employing the anti-angiogenic effect of small-molecule PKIs to investigate the aforementioned hypothesis. A series of subtly designed *in vitro* or *in vivo* experiments with any of these nine small molecules may lead to potential success in verifying our hypothesis (Table 2). The potential advantage of this strategy is that the mechanism of drug action of these nine small molecules has been well studied. Meanwhile, their safety and effectiveness have already been confirmed by strict clinical trials.

#### 8.2.1. Two strongly-suggested anti-angiogenic small molecules

For pilot investigations, we highly recommend axitinib and tivozanib, because these two anti-angiogenic molecules reveal more target specificity toward VEGFRs and PDGFRs than the other seven molecules. Axitinib is an inhibitor of VEGFR1, VEGFR2, VEGFR3, PDGFRα, and PDGFRβ, approved by the U.S. FDA in 2012 for the second-line therapy for advanced renal cell carcinoma (RCC) (124–127). Similarly, tivozanib is an inhibitor of VEGFR1, VEGFR2, VEGFR3, and PDGFRβ, approved by the U.S. FDA in 2021 for the third-line therapy for advanced RCC (132–134). Both axitinib and tivozanib own higher inhibitory activity against VEGFRs than PDGFRs (125, 134). One characteristic of primary and advanced RCCs is their resistance to chemotherapy and radiotherapy (138, 139). It is noteworthy that the underlying mechanism of axitinib and tivozanib for treating advanced RCC is inhibiting tumor angiogenesis rather than directly killing the cancer cell.

#### 8.2.2. Proper selection for animal species and nerve models

Animal experiments on peripheral nerves serve to answer questions that cannot be investigated in the human species because challenging is to directly study the basic knowledge about cellular responses and molecular events from clinical practices on human nerves. However, before asking a specific question concerning peripheral nerve injury, it is essential to consider whether an animal model is suitable to provide a reasonable answer (36). The sciatic nerves of rats have been the most popular subject for experimental neuromas (140). Unfortunately, the ends of proximal stumps of transected rat sciatic nerves did not form classic terminal neuromas resembling human ones (141). In this sense, higher species may be more appropriate, such as dogs, monkeys, baboons, and chimpanzees (46, 142). Hence, for pilot research, the suture line neuroma model of rodents may be more rational than the terminal neuroma model (30, 31).

#### 8.2.3. Administration route, dosage, and timing

Previously, our research team demonstrated that the subcutaneous injection of an axitinib-dimethyl sulfoxide (DMSO)-saline solution (10 mg axitinib per 1 kg animal weight) twice per day successfully inhibits angiogenesis within the choke zones of the rat vascular delay flap model (143). Therefore, for the exploratory study, we recommend the topical administration of small-molecule PKI-DMSO solution with a single dose (10 mg PKI per 1 kg animal weight) at a time on the nerve trunk immediately after nerve transection injury.

## 9. Conclusion

In this review, we put forward the concept of nerve trunk healing and roughly divide this process into three biological phenomena: angiogenesis, nerve fiber regeneration, and scarring (Figure 4). Subsequently, we infer that nerve trunk healing and neuroma formation are two sides of the same coin. Furthermore, we analyze the potential relationship between the three processes based on literature about nerve transection injuries in animal models. Briefly, angiogenesis at the nerve transection site is sufficient and necessary for nerve fiber regeneration and may also be necessary for scarring; a positive correlation exists between angiogenesis and nerve fiber regeneration in the early phase while a negative correlation exists between scarring and nerve fiber regeneration in the late phase (Figure 6).

Nowadays, no one can deny that the best treatment for a neuroma is to prevent its formation (45). Inspired by the molecular knowledge from the field of tumor angiogenesis, we hypothesize that the anti-angiogenic strategy suppresses neuromas. Innovatively, we recommend employing nine anti-angiogenic small-molecule PKIs approved by U.S. FDA to test our hypothesis and provide feasible protocols (Figure 7) (Table 2).

Investigating nerve trunk healing as well as neuroma formation with anti-angiogenic small-molecule PKIs may open new avenues in the field of peripheral nerve injury. First, applying anti-angiogenic small-molecule PKIs at the nerve transection sites may facilitate the understanding of the relationship between angiogenesis and scarring during nerve trunk healing, which is still unknown in the literature. Second, if the anti-angiogenic effect of small-molecule PKIs contributes to the suppression of neuromas, the topical application of these molecules may facilitate the treatment of neuromas as adjuvant therapy. Third, if neuromas are successfully suppressed by these U.S. FDA-approved molecules in animal models, preclinical studies and clinical trials may be designed for the repurposing of these drugs for neuroma prevention or treatment.

## Author contributions

XG contributed to the conception and design of the study. D-XH and M-XY wrote the first draft of the manuscript. XG, D-XH, M-XY, Z-MJ, and MC wrote the second draft of the manuscript. XG and D-XH reviewed and edited the third and fourth draft of the manuscript. XG, D-XH, KC, and Y-XZ searched and prepared the references. D-XH, Z-MJ, and MC summarized the tables. D-XH, KC, and Y-XZ designed the figures. All authors have read and approved the submitted version.

## Conflict of interest

The authors declare that the research was conducted in the absence of any commercial or financial relationships that could be construed as a potential conflict of interest.

## Publisher’s note

All claims expressed in this article are solely those of the authors and do not necessarily represent those of their affiliated organizations, or those of the publisher, the editors and the reviewers. Any product that may be evaluated in this article, or claim that may be made by its manufacturer, is not guaranteed or endorsed by the publisher.
